# *In Vivo* Biocompatibility of an Ionic Liquid-protected Silver Nanoparticle Solution as Root Canal Irrigant

**DOI:** 10.22037/iej.v13i3.17386

**Published:** 2018

**Authors:** Mohammadreza Nabavizadeh, Yasamin Ghahramani, Abbas Abbaszadegan, Akram Jamshidzadeh, Peyman Jenabi, Alireza Makarempour

**Affiliations:** a *Oral and Dental Disease Research Center, Dental School, Shiraz University of Medical Sciences, Shiraz, Iran; *; b *Department of Endodontics, Dental School, Shiraz University of Medical Sciences, Shiraz, Iran; *; c *Pharmaceutical Sciences Research Center, Shiraz University of Medical Sciences, Shiraz, Iran; *; d *Pathologist, Shiraz, Iran*

**Keywords:** Biocompatibility, Root Canal Irrigant, Silver Nanoparticle

## Abstract

**Introduction:**

The aim of this study was to assess the biocompatibility of positively charged imidazolium-based ionic liquid-protected nanosilver solution (AgNPs) root canal irrigant.

**Methods and Materials:**

Eighteen male 4- to 5-month old Sprague-Dawley rats, weighing 200-300 gr were selected and randomly divided into 5 groups: Normal saline 0.9% (group 1), 5.25% NaOCl (group 2), 2.5% NaOCl (group 3), 2.0% chlorhexidine solution (group 4) and AgNPs at 5.7×10^-8^ M/L (group 5) were randomly injected in 5 sites of dorsal skin of each rat. Tissue inflammatory reaction were evaluated histopathologically after 2 h, 48 h and 14 days. Statistical analysis was done with SPSS version 21 and the Kruskal-Wallis H and Dunn tests were used to find statistically significant differences. The level of significance was set at 0.05.

**Result:**

All solutions irritated the highest tissue response after 48 h. Group 1 showed lower inflammatory response compared to groups 2 and 4 (*P*<0.05). Group 2 displayed higher inflammatory response in comparison with group 5 (*P*<0.05). Tissue reaction to group 5 was not more severe than the reaction to group 3 or 4. It also would irritate less inflammatory response compared to group 2 (*P*<0.05).

**Conclusion:**

Comparing with NaOCl and CHX, it is possible to label AgNPs as a tissue compatible agent.

## Introduction

One of the purposes of root canal therapy is to eradicate debris, tissue remnants and bacteria from the root canal system [1, 2]. Due to anatomic complexity of the root canal system, bacteria which shelter deep inside the dentinal tubules cannot be eliminated even after accurate mechanical instrumentation [3, 4]. Thus, application of chemical irrigant is necessary for eradication of remained infected tissues [5, 6]. Sodium hypochlorite (NaOCl) as one of the most popular intra-canal irrigants has some shortcoming such as bad odor, inability in removing the inorganic parts of smear layer and being harmful to periapical tissues in case of entering the periradicular space. Chlorhexidine gluconate (CHX) is another commonly used intra-canal irrigant but it cannot dissolve the inorganic and organic components despite its high antibacterial potency [7, 8].

Recent studies have focused on nanosilver (NS) products as possible alternative for endodontic irrigants [9, 10]. The antibacterial effect of NS solution has been evaluated previously; it is revealed that imidazolium based AgNPs had strong bactericidal efficacy, in very low concentrations compared to NaOCl and CHX [11, 12]. It is also indicated that NS is effective against antibiotic resistant bacteria [13].

Biocompatibility of NS is still a controversial topic. Some evidence warn about the adverse effects of NS on human health *via* non-specific oxidative destruction and silver inducing cytotoxicity [14, 15]. From the other point of view, Erik *et al.* [16] displayed that NS was not cytotoxic for mouse fibroblasts and human periodontal ligament stem cells. Also Takamiya *et al.* [17] proved the cytocompatibility of NS on L929 in low concentrations. The positively charged imidazolium-based ionic liquid-protected NS (AgNP) showed high level of antibacterial efficacy against *Enterococcus faecalis.* It is revealed that AgNP has lower cytotoxic effect on L929 in comparison to NaOCl and CHX [10]. Since patient safety is an important concern for clinicians and considering the lack of studies on the biocompatibility of AgNP solution, this study evaluated the compatibility of AgNP with vital tissue in comparison with 2.5% NaOCl, 5.25% NaOCl and 2% CHX.

**Figure 1:**
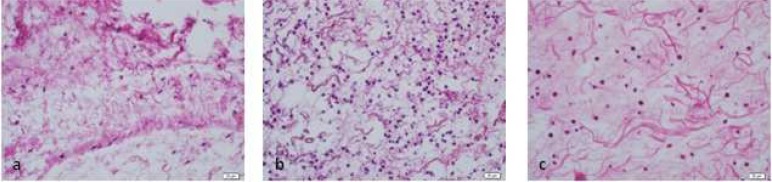
Tissue reaction to 5.7×10^-8^ M/L AgNPs (H&E ×400); *A)* 2 h after injection; *B)* 48 h after injection; *C)* 14 d after injection

**Figure 2.**
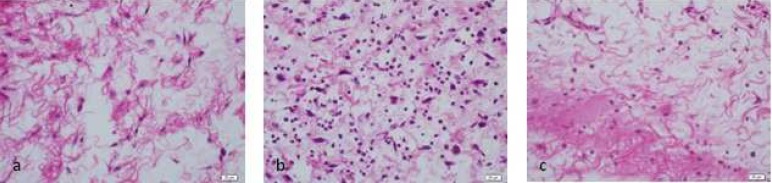
Tissue reaction to 2.5% NaOCl (H&E 400×); *A)* 2 h after injection; *B)* 48 h after injection; *C)* 14 d after injection

**Figure 3:**
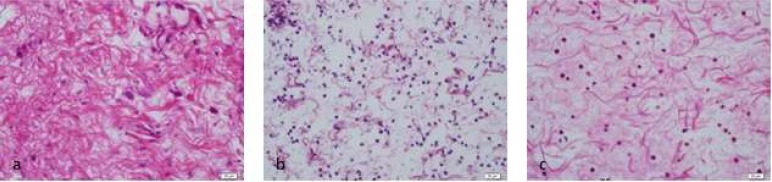
Tissue reaction to 2% CHX (H&E ×400); *A)* 2 h after injection; *B)* 48 h after injection; *C)* 14 d after injection

**Figure 4.**
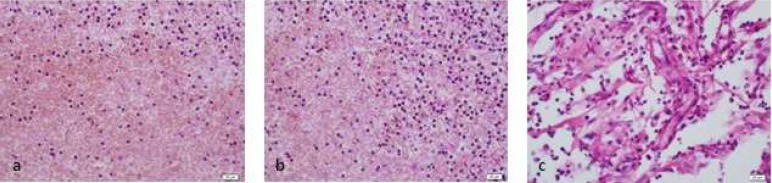
Tissue reaction to 5.25% NaOCl (H&E ×400); *A)* 2 h after injection; *B)* 48 h after injection; *C)* 14 d after injection

## Materials and Methods

Eighteen male 4- to 5-month old Sprague-Dawley rats, weighing 200-300 gr were selected for the present study. Animals were housed in plastic cages over hardwood bedding. The temperature was set at 24^◦^C along with a 40% of relative humidity. The rats were allowed ad-libitum access to a normal standard chow diet and water. Animals received human care and all the experiments were performed in conformity with the guidance for care and use of experimental animals approved by a local ethic committee in Shiraz University of Medical Sciences, Shiraz, Iran (#95-01-03-12208).

Animal tissue reaction was evaluated against irrigants as follows; group 1, normal saline 0.9% (control group); group 2, 5.25% NaOCl (Cerkamed, Pawłowski, Poland); group 3, 2.5% NaOCl (Cerkamed, Pawłowski, Poland); group 4, 2.0% chlorhexidine solution (Cerkamed, Pawłowski, Poland) and group 5, AgNPs at 5.7×10^-8^ M/L which was prepared according to the method suggested by Abbaszadegan *et al.* [9]. 

The animals received anesthesia with xylazine (10 mg/kg) and ketamine (25 mg/kg) and the dorsal skin was shaved, cleaned and disinfected with 10% iodine solution. By a transplant template, 5 circles were marked on dorsal of each rat, leaving 3 cm between the circles. Then 0.1 mL of each irrigant was injected subcutaneously into the circles. Tissue reaction was evaluated in three period times after the injections as following; 2 h, 48 h and 14 days. Rats were sacrificed at each period by overdose of xylazine/ketamine solution. The dorsal skin tissue with the thickness of 2-3 mm was excised with a surgical blade and fixed in 10% buffered formalin. The tissue processing and histopathological slide preparation were performed according to the procedure introduced by Gomes-Filho’s [18]. The histopathological sections were quantitatively evaluated and the inflammatory cells such as lymphocytes, plasmocyte, polymorpho-nuclear leukocytes (PMN), macrophages and giant cells were counted *via* a light microscope (Olympus Corporation Ina Plant, Ina, Japan) under ×400 magnification. An average of the inflammatory cells for each group was obtained from 5 separate areas in histologic sections. Tissue reaction of each irrigant was assessed *via* sum of inflammatory cells existing in its corresponding histopathologic sections [14]. According to Gomes and Filho, tissue reaction was scored as following: 0- none or few inflammatory cells define as no reaction; 1- less than 25 cells define as mild reaction; 2- between 25 and 125 cells define as moderate reaction and 3- 125 and more cells define as severe reaction [14, 19]. 

**Table 1:** Tissue inflammatory reaction to irrigants in different periods of time. (mild: mild inflammation, moderate: moderate inflammation, severe: severe inflammation and no: no inflammation

	**Time**			
**Group**	**Inflammatory Reaction**	**2 h**	**48 h**	**14 d**
**0.9% sterile saline**	No Mild Moderate Severe	1 5 0 0	1 5 0 0	6 0 0 0
**5.25% NaOCl**	No Mild Moderate Severe	0 0 0 6	0 0 0 6	0 0 6 0
**2.5% NaOCl**	No Mild Moderate Severe	0 5 1 0	1 0 5 0	0 0 6 0
**2% CHX**	No Mild Moderate Severe	0 4 2 0	0 0 6 0	1 0 5 0
**5.7×10** ^-8^ ** M/L AgNPs**	No Mild Moderate Severe	0 6 0 0	0 0 6 0	0 0 6 0

**Table 2.** Comparison of tissue reaction to studied solutions at different time periods. In each time median values with at least a common capital letter were not statistically different. In each group median values with at least a common lower letter were not statistically different. (*Kruskal-Wallis H test

**Groups**	**Time**			***P*** **-value** ^*^
	**2 h**	**48 h**	**14 d**	
**0.9% sterile saline**	5 ^A,ab^	9 ^A, a^	0 ^A,b^	0.002
**5.25% NaOCl**	80 ^B, a^	150 ^B, b^	87 ^B, ab^	0.002
**2.5% NaOCl**	20 ^AB, a^	100 ^AB, b^	29 ^AB, a^	0.001
**2% CHX**	21 ^BC, a^	110 ^BC, b^	34 ^BC, ab^	0.001
**5.7×10** ^-8^ ** M/L AgNPs**	14 ^AC, a^	100 ^AC, b^	27 ^AC, ab^	<0.001
***P*** **-value** ^*^	<0.001			

Data were expressed as mean and standard deviation (SD) and analyzed using Statistical Package for the Social Sciences software (SPSS, version 21.0, Chicago, IL, USA). The Kruskal-Wallis H and Dunn’s tests were used to find statistical significant differences between different irrigants. A *P*-value less than 0.05 was considered as statistically significant.

## Results

***Tissue reaction to irrigants after each period of time***

According to data presented in Table 1, in group 1 (control group) inflammatory reaction to 0.9% sterile saline solution was mild. In group 2, inflammatory reaction to 5.25% NaOCl solution was moderate to severe in different periods of time. In group 3, 2.5% NaOCl solution caused mild to moderate reaction. In group 4, inflammatory reaction to 2% CHX solution was mild to moderate and finally in group 5, inflammatory reaction to 5.7×10^-8 ^M/L AgNPs solution was mild to moderate (Figures 1 to 4).

Considering all groups in mentioned time periods, statistically significant differences in tissue inflammatory response were found that are reported in Table 2. No statistically significant differences were reported among different studied groups at any time periods except for the following ones; group 1 showed lower inflammatory response comparing to groups 2 and 4 (*P*<0.05). In addition, group 2 displayed higher inflammatory response compared to group 5 (*P*<0.05).

## Discussion

Considering lack of studies on the biocompatibility of AgNP solution as an intracanal endodontic irrigant, this study designed to evaluate the compatibility of AgNP with vital tissue comparing with 2.5% NaOCl, 5.25% NaOCl and 2% CHX. An optimal endodontic irrigant should have the ability to dissolve tissue, eliminate resistant bacteria and does not induce inflammatory reaction in case of inadvertent extrusion [18, 20, 21]. The bactericidal potency of silver has been demonstrated; also silver reduces bacterial adhesion and prevents biofilm formation [22]. Reducing the silver particle size to the range of nanoparticles can lead to a higher antibacterial potency [9]. Antibacterial studies have demonstrated that silver nanoparticles are effective against a broad range of gram positive and gram negative bacteria and their antimicrobial activity is comparable or better than the broad spectrum antibiotics [23]. Although, the exact mechanism of AgNPs cytotoxicity is not still clear, previous studies reported that oxidative stress, mitochondrial dysfunction, DNA damage and cytokine induction may be the main cause of their cytotoxicity [24, 25].

An *in vitro* study has demonstrated that 5.7×10^-8^ M/L AgNPs was effective against bacteria, especially *E. faecalis *whilst it had only a mild cytotoxicity to L929 fibroblasts [10]. It has been demonstrated that the intensity of NPs toxicity is concentration dependent [25] and also related to shape, size and surface charge of these particles [26, 27], so in the current study the tissue reaction of AgNPs irrigant with similar concentration and characterization used in the study by Abbaszadegan *et al*. [10] was compared with the tissue response to 2.5% NaOCl and 2% CHX in different periods.

In the present study, 0.9% sterile saline as control group created only a mild inflammation in first 48 h. This finding is in agreement with other studies [18, 20, 21] considering saline as a biocompatible material. The early tissue responses of all tested irrigants except 5.25% NaOCl have demonstrated mild inflammation. The moderate inflammation of 5.25% NaOCl might be due to its innate caustic effect in higher concentrations [28]. 

It was found that AgNPs had no statistically significant difference in comparison with 2.5% NaOCl and 2% CHX in promoting tissue reaction at all periods of time. In addition, AgNPs was significantly more tolerated by the tissue than 5.25% NaOCl at 2 h, 48 h and 14 days 

AgNPs showed mild inflammatory response at 2 h and increased to moderate reaction until 14 days. Gomes-filho *et al.* [14] have compared the biocompatibility of AgNPs with 2.5% NaOCl and found that AgNPs displayed moderate inflammatory reaction at day 7 and day 14, which seems to be consistent with the present study. Although, Chen *et al.* [29] after implanting AgNPs into the back of rat observed sever inflammation up to 180 days. The particle size in the Chen’s study was smaller than 100 nm, which was significantly larger than particle size in our study (5-10 nm). In the at 48 h and remained elevated until the end of the test period, which is in accordance with some other studies reporting that the number of inflammatory cells remained high at the site treated with 5.25% NaOCl after 14 days [20, 21, 30]. Also Yesilsoy *et al.* [21] has shown that 5.25% NaOCl could irritate periapical tissues and induce a foreign body granuloma two weeks after the exposure. The highly irritative ability of NaOCl is concentration-, volume- and temperature-dependent. In addition, the pressure of injection can exacerbate the conditions [31]. NaOCl would induce hemorrhage immediately after getting into contact with vital tissue [32]. Besides, it would rapidly cause tissue oxidization, [31] which could consequently result in hemolysis and loss of cellular protein [32]. Consequently, evidences do not suggest 5.25% NaOCl as a safe irrigant for endodontic purposes, especially in case of perforations and open apices.

The 2% CHX and 2.5% NaOCl presented mild inflammatory reaction at 2 h. The inflammation increased to moderate after 48 h. After 14 days, a decrease in the number of inflammatory cells was found which could be the result of connective tissue repair process. This Also accords with the studies evaluating 0.12% CHX as an endodontic irrigant which reported that the mean number of inflammatory cells dropped after 14 days [20, 21]. Also Gomes-filho *et al.* [18] showed that 2.5% NaOCl induced a moderate inflammatory reaction at day 7 and day 14, which is consistent with the present study. 

The tested materials injected subcutaneously at the dorsum of rats, using the method described by Gomes-filho *et al*. [18]. In this method, it was not needed to incise the skin surgically and the experimental solutions would be directly in close contact to connective tissue cells. The difficulty in finding the injection site on skin after a few days and the diffusion of the injected solution from the application site would be some limitations of this study. Further studies on the biocompatibility of AgNPs would be essential to ascertain the safety for clinical application. The authors would also suggest studying the tissue reactions for longer periods regarding any possible dissolution of nano-silver particles into more toxic ionic form.

## Conclusion

This study concluded that, AgNPs solution was biocompatible in comparison to 2.5% NaOCl and 2% CHX, but more future studies are needed to confirm the observed results.
